# Characteristics and outcomes of anterior mediastinal cystic lesions diagnosed on chest MRI: implications for management of cystic lesions

**DOI:** 10.1186/s13244-022-01275-8

**Published:** 2022-08-17

**Authors:** Jooae Choe, Sang Min Lee, Yura Ahn, Chu Hyun Kim, Joon Beom Seo, Ho Yun Lee

**Affiliations:** 1 Department of Radiology and Research Institute of Radiology, Asan Medical Center, University of Ulsan College of Medicine, 88 Olympic-ro 43 Gil, Songpa-gu, Seoul, 05505 Korea; 2 Department of Radiology and Center for Imaging Science, Samsung Medical Center, Sungkyunkwan University School of Medicine (SKKU-SOM), 81 Irwon-Ro, Gangnam-Gu, Seoul, 06351 Korea; 3 Department of Health Sciences and Technology, SAIHST, Sungkyunkwan University, Seoul, 06351 Korea

**Keywords:** Magnetic resonance imaging, Mediastinal cyst, Thymoma, Thymus gland

## Abstract

**Background:**

Chest MRI is a useful diagnostic modality for the evaluation of anterior mediastinal lesions but the outcomes of anterior mediastinal cystic lesions diagnosed on chest MRI are unclear.

**Methods:**

In this multicenter retrospective study, patients who underwent contrast-enhanced chest MRI in two tertiary centers to assess anterior mediastinal cystic lesions were included after excluding overt solid tumors and thymic hyperplasia. Anterior mediastinal cystic lesions were classified into two categories: probable (simple) cyst or indeterminate lesion (complex cyst). Size and imaging features of lesions during follow-up were evaluated and clinical outcomes were assessed.

**Results:**

A total of 204 patients (mean age, 59 ± 11 years; M:F = 111:93) were studied; 186 (91.2%) were classified as probable cysts and 18 (8.8%) as indeterminate lesions on MRI. Among patients with probable cysts and more than 2 years of follow-up, lesion size was unchanged in 39.6% (36/91), decreased in 16.5% (15/91), and fluctuated in 8.8% (8/91). All patients who underwent surgery were confirmed cysts. None developed mural nodules or irregular wall thickening, suspicious for malignancy during follow-up. In patients with indeterminate lesions, 16.7% (3/18) had pathologically confirmed thymoma and 44.4% (8/18) had proven cysts. Follow-up numbers and intervals after MRI in patients with probable cysts were variable among physicians and institutions in clinical practice (*p* < 0.05) but more than half were followed for up to 2 years in two centers.

**Conclusion:**

Diagnosing anterior mediastinal cysts using MRI is reliable. MRI-based management of anterior mediastinal lesions may reduce the number of unnecessary follow-ups and surgeries.

**Supplementary Information:**

The online version contains supplementary material available at 10.1186/s13244-022-01275-8.

## Key points

- Diagnosing anterior mediastinal cysts using MRI is reliable.
- None of probable cysts on MRI turned out to be tumors.
- For indeterminate lesions, short-term follow-up can be recommended prior to surgery.
- MRI-based management may reduce the unnecessary follow-ups and surgeries.

## Background

With the increasing use of chest CT in routine clinical practice and cancer screening, asymptomatic anterior mediastinal lesions are being detected more frequently. The prevalence of incidental anterior mediastinal lesions is reported to be 0.5–0.9% [1–3]. The most prevalent benign disease for anterior mediastinal lesions is a thymic cyst, and malignancies such as thymic epithelial tumors are a primary concern for incidental anterior mediastinal lesions. Diagnosing asymptomatic anterior mediastinal lesions based on CT findings is often challenging, as the lesions can lack distinguishable CT features owing to their small size, ovoid shape, and CT attenuation higher than 20 Hounsfield units [2, 4]. Although thymoma grows with a wide range of volume doubling times [5, 6], thymic cyst also can show growth on follow-up [7]. Moreover, because of their location, small anterior mediastinal lesions are not always readily accessible by percutaneous biopsy, occasionally causing patients to undergo unnecessary thymectomy for ultimately benign disease.

Chest magnetic resonance imaging (MRI) is a useful diagnostic modality that can help distinguish cystic from solid thymic lesions that are indeterminate on CT [8, 9]. The International Thymic Malignancy Interest Group (ITMIG) suggested the use of MRI for work up and also follow-up of suspected anterior mediastinal cystic lesion [10]. Reported diagnostic accuracy in differentiating non-neoplastic cyst from malignant tumor using chest MRI is 71–91% [9, 11, 12]. However, the guidelines did not clearly describe how to manage suspected anterior mediastinal cystic lesions on MRI in terms of follow-up interval or duration. Recently, Ackman et al. demonstrated the longitudinal change of unilocular thymic cysts defined by MRI, which showed changes in size and morphologic features without definite malignant transformation [7]. However, we also occasionally encounter multilocular cystic lesions or cystic lesions with nodular or eccentric wall thickening on imaging which are also clinically significant for patient management but not evaluated in the previous study. Therefore, we aimed to evaluate the follow-up outcome in patients with anterior mediastinal cystic lesions stratified based on MRI findings, and assess the clinical implication of chest MRI for the management of anterior mediastinal cystic lesions.

## Materials and methods

This retrospective study was approved by the institutional review boards, and the requirement for informed consent was waived (IRB Numbers: 2021-0402, 2021-02-156).

### Study design and patients

**Fig. 1:**
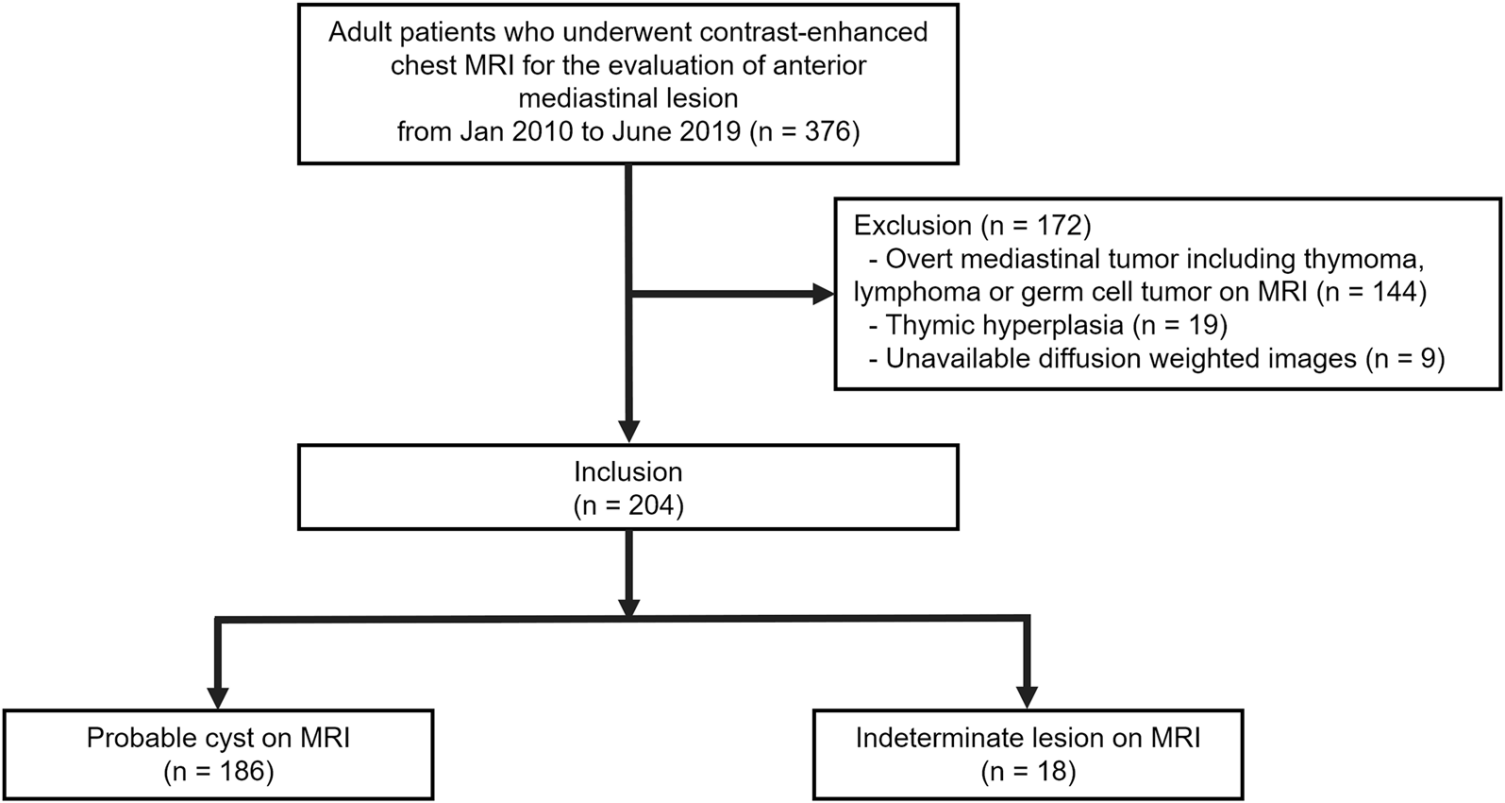
Flowchart of study inclusion

In this multicenter study, consecutive patients who underwent contrast-enhanced chest MRI for the evaluation of anterior mediastinal lesion manifesting as a cystic lesion were included from January 2012 to June 2019 at Asan Medical Center (institution A) and from January 2010 to June 2019 at Samsung Medical Center (institution B). All MRI examinations were performed to evaluate incidental anterior mediastinal lesions detected on CT. Exclusion criteria were overt solid tumor on MRI that showed solid enhancement of a nodule or mass, thymic hyperplasia, and poor image quality or at least one sequence unavailable among T2-weighted, T1-weighted (pre- or postcontrast), or diffusion-weighted images (DWIs) (Fig. 1) [10, 13].

The medical record of each patient was reviewed, and the clinical information (patient age, sex, reason for imaging follow-up, follow-up numbers, and intervals after chest MRI) and pathologic findings if the cystic lesion was resected were recorded.

### Imaging protocols and scanners

Imaging studies including baseline and follow-up chest CT scans and MRI were performed with multidetector CT and MRI from different vendors and two magnet strengths (1.5 and 3.0 T). MRIs from institution A (*n* = 170) were obtained with either 3.0 T scanner (MAGNETOM Skyra, Siemens Healthineers, Erlangen, Germany; *n* = 137) or 1.5 T scanner (MAGNETOM Avanto, Siemens Healthineers, Erlangen, Germany; *n* = 33). MRIs from institution B (*n* = 33) were obtained with 3.0 T scanners from different vendors, either Philips (Ingenia, Philips Healthcare, Best, the Netherlands; *n* = 13) or Siemens (MAGNETOM Skyra, Siemens Healthineers, Erlangen, Germany; *n* = 20).

The MRI protocol included at least the following sequences: (1) axial T2-weighted images with fat-suppression, (2) pre- and post-contrast axial T1-weighted images with fat-suppression, and (3) DWIs with corresponding apparent diffusion coefficient (ADC) maps. T2-weighted imaging was performed mostly with breath-hold electrocardiography-gated double inversion recovery T2-weighted imaging. Electrocardiography-gating was applied to obtain better and reliable image quality with high-signal and high-resolution images of the lesion of interest. Images were mostly obtained before and after dynamic administration of gadolinium, predominantly with breath-hold three-dimensional ultrafast gradient-echo fat-saturated T1-weighted imaging (93.6%, 191/204) and occasionally with fat-saturated turbo spin-echo imaging (6.4%, 13/204). Breath-hold DWIs were obtained with b values of 0, b values of 100 or 500 s/mm^2^, and b values of 700 or 1000 s/mm^2^. The details of MRI protocols are demonstrated in the Additional file 1: Tables S1 and S2.

### Image analysis

All imaging studies were reviewed by two thoracic radiologists (J.C. and S.M.L., with 5 years’ and 10 years’ experience in thoracic radiology, respectively) who were blinded to clinical information. Diametric measurements were performed manually on multiplanar images (axial, sagittal, or coronal) with the largest diameter using electronic calipers. The single largest diameter at each examination was recorded, and a measurable change in the size of anterior mediastinal lesion was defined as a decrease or increase in more than 2.5 mm in the largest size between the two examinations [14, 15].

**Fig. 2:**
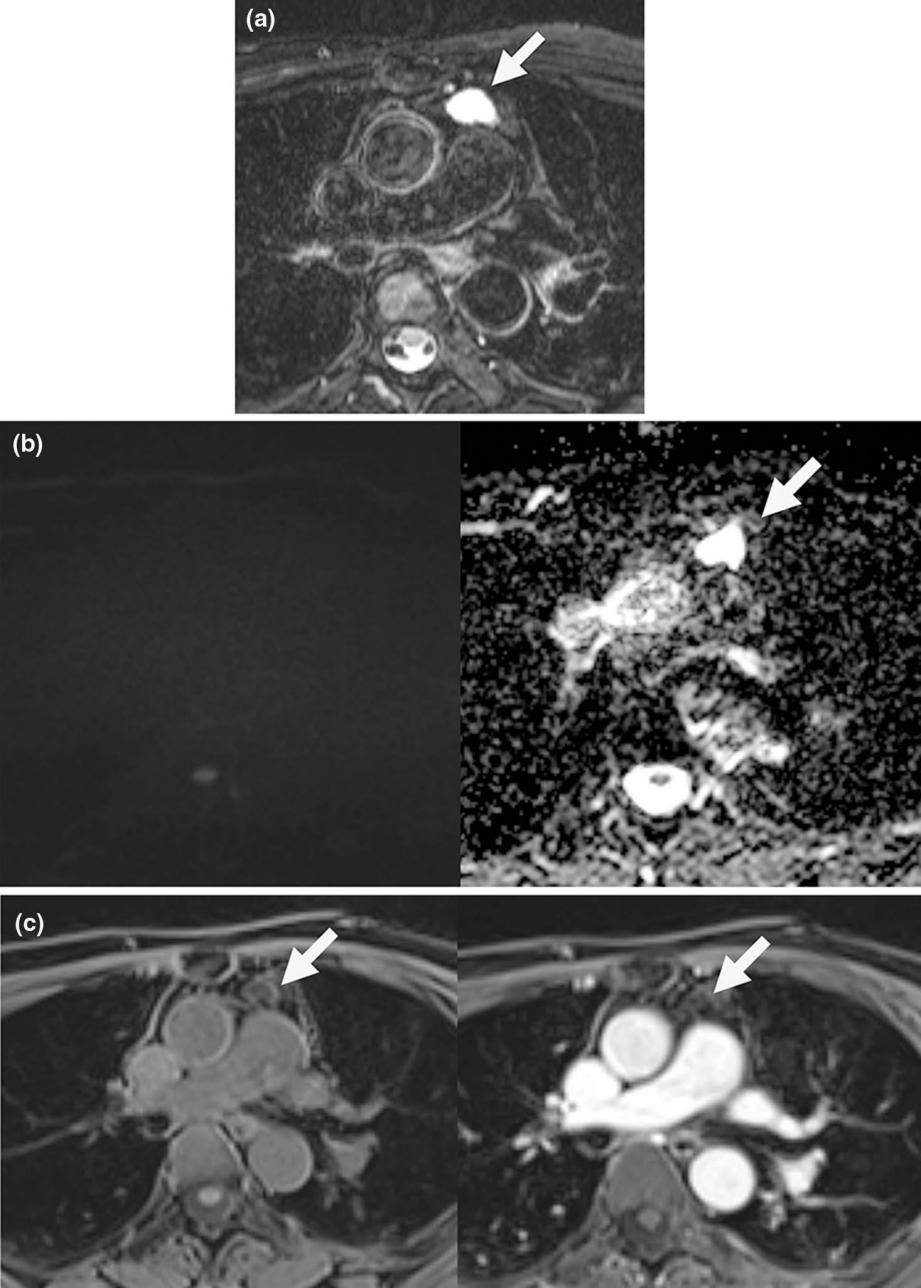

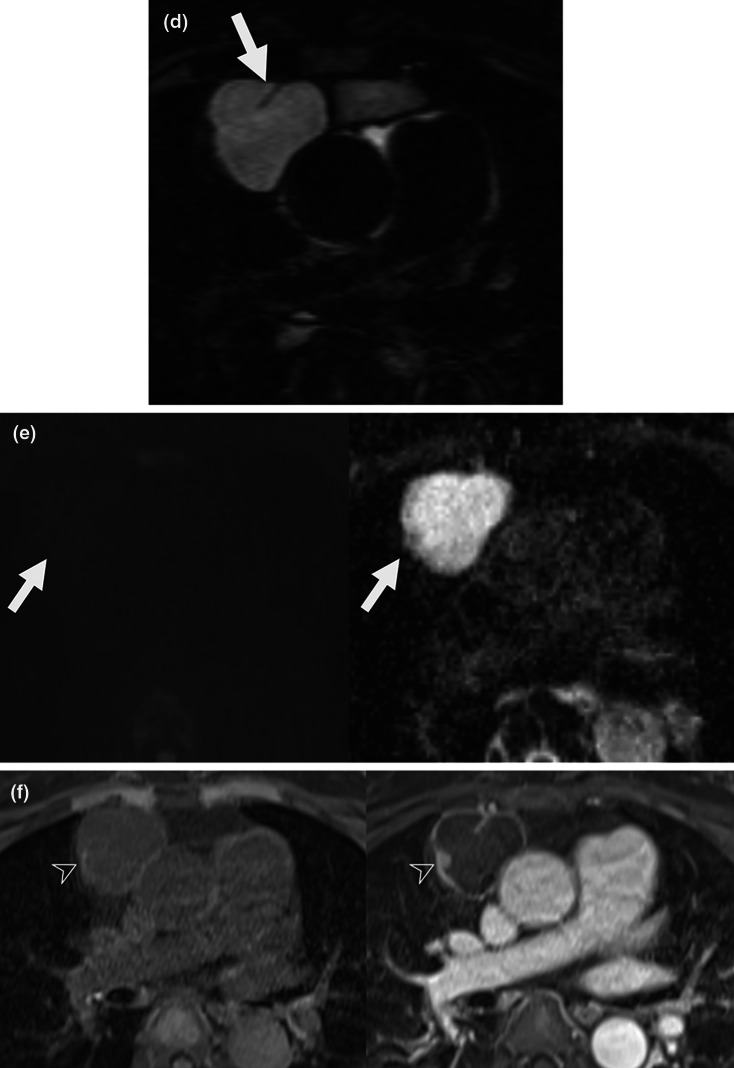
MR findings of probable cyst (simple cyst, category 1) and indeterminate lesions (complex cyst, category 2). Chest MRI in a 50-year-old female (**a**–**c**) shows 22-mm well-defined, ovoid, and unilocular lesion (arrows) in the anterior mediastinum showing homogeneous high signal intensity on T2-weighted image (**a**) and no remarkable diffusion restriction on high b-value (b1000) diffusion-weighted image and high apparent diffusion coefficient (ADC) value (**b**). The lesion shows intermediate signal intensity on pre-contrast T1-weighted image, and no remarkable enhancement on post-contrast T1-weighted image (**c**). The lesion can be classified as a probable cyst based on chest MRI findings. Chest MRI in a 65-year-old female (**d**–**f**) shows a 43-mm lobulated and multilocular lesion in the anterior mediastinum showing internal high signal intensity on T2-weighted image (arrow, **d**) and no remarkable diffusion restriction (**e**). The lesion shows intermediate signal intensity on pre-contrast T1-weighted image and diffuse enhancement along cyst wall, and septa with a nodular solid enhancement attached to the right lateral side of the cyst wall on post-contrast T1-weighted image (arrowhead, **f**). The lesion can be classified as an indeterminate lesion based on chest MRI findings. The patient underwent thymectomy one month after chest MRI. On gross pathology, the cystic mass was well-defined and multiseptated with a focal solid portion and sebaceous material. The cystic mass was compatible with the thymic cyst on histopathology

Imaging features of anterior mediastinal lesions were assessed on MRI, including internal signal intensity on T2-weighted image and pre-contrast T1-weighted image relative to muscle, the presence of diffusion restriction, and the presence of contrast-enhancement of the lesion (smooth thin wall enhancement, nodular enhancing solid portion, eccentric thickening of septa or wall with enhancement). Signal intensities of lesions were measured on representative images with regions of interest covering the entire lesion. Fat-saturated axial pre-contrast T1-weighted images and breath-hold electrocardiography-gated double inversion recovery T2-weighted images were preferentially used for evaluation of T1 and T2 signal intensities, respectively. Based on MRI findings, all lesions were classified into two categories: category 1, probable cysts (or simple cyst) vs. category 2, indeterminate lesion (complex cyst) (Fig. 2). The MRI criteria for probable cyst (or simple cyst) included the following features: 1) no eccentric wall thickening or nodular enhancement except smooth thin wall enhancement, 2) no diffusion restriction, and 3) high signal intensity on T2-weighted image. An indeterminate lesion (complex cyst) was defined when the lesion did not satisfy the above findings. Confidence level of the diagnosis was also rated for probable cysts by 3-point scale (high, intermediate, and low).

For the evaluation of follow-up outcomes, the change in size of lesion and development of new nodular solid portion or irregular wall thickening on both CT and MRI were evaluated and events of surgery and incidence of confirmed tumor were recorded.

### Statistical analysis

Descriptive statistics were demonstrated as mean ± standard deviation, median with interquartile range, or numbers with percentage. Variables were compared using the Fisher’s exact test, Chi-square test or Mann–Whitney U test, as appropriate. Follow-up strategies among physicians and between institutions were compared using the Kruskal–Wallis test or Mann–Whitney U test. All statistical analyses were performed using SPSS software (version 19.0, IBM Corp., Armonk, NY, USA). *P* values < 0.05 were considered to indicate statistical significance.

## Results

### Patient characteristics

A total of 204 patients (mean age 59 ± 11 years; M: F = 111:93) underwent chest MRI. Among these patients, 186 (91.2%) were classified as probable cyst and 18 (8.8%) were classified indeterminate lesion on MRI. The median long-axis diameter of the lesion on MRI was 24 mm (interquartile range [IQR], 18–38; range, 7–113). The patient characteristics of the study population are presented in Table 1. The total follow-up duration from initial to last follow-up imaging in total patients was mean 2.4 years and median 1.7 years (range, 0–12.3 years). The follow-up duration was median 1.9 years (range, 0–12.3 years) for probable cyst and median 0.3 years (range, 0.1–5.5 years) for indeterminate lesions. Except for patients who underwent immediate surgery after chest MRI, the total follow-up duration was median 2.0 years (range, 0–12.3 years) in all patients, median 2.0 years (range, 0–12.3 years) in patients with probable cyst, and median 1.0 years (range, 0.1–5.5 years) in patients with indeterminate lesions.

**Table 1:** Patient characteristics of the study population

	Total (*n* = 204)	Probable cyst (*n* = 186)	Indeterminate lesion (*n* = 18)
Age, years^a^	59.2 ± 11.1	59.4 ± 10.8	56.5 ± 14.1
Sex			
Male	111 (54.4)	102 (54.8)	9 (50.0)
Female	93 (45.6)	84 (45.2)	9 (50.0)
Size, mm^b^	24 (18–38)	24 (18–33)	39 (22–54)
Follow-up duration, year^b^	1.7 (0.5–3.3)	1.9 (0.6–3.3)	0.3 (0.2–1.3)
Range	0–12.3	0–12.3	0.1–5.5

Unless indicated otherwise, data are percentages in parentheses

^a^Data are means ± standard deviations, with ranges in parentheses

^b^Data are median with interquartile ranges in parentheses

### Imaging characteristics and outcome of probable cysts on chest MRI

**Fig. 3:**
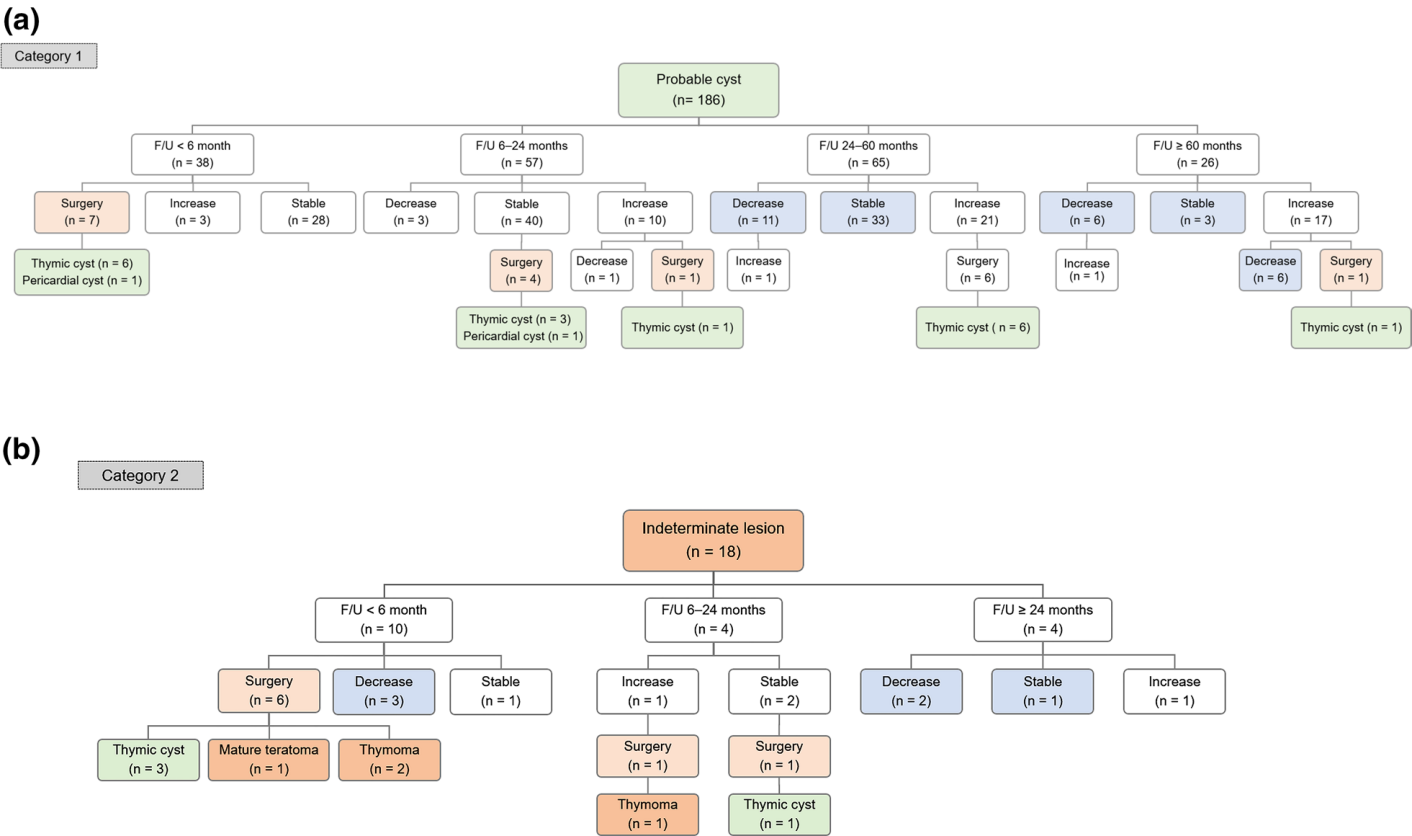
Follow-up outcomes of anterior mediastinal cystic lesions—stratified based on MRI findings and follow-up outcomes. Follow-up outcomes in patients with probable cyst (**a**) and indeterminate lesion (**b**) on chest MRI

**Fig. 4:**
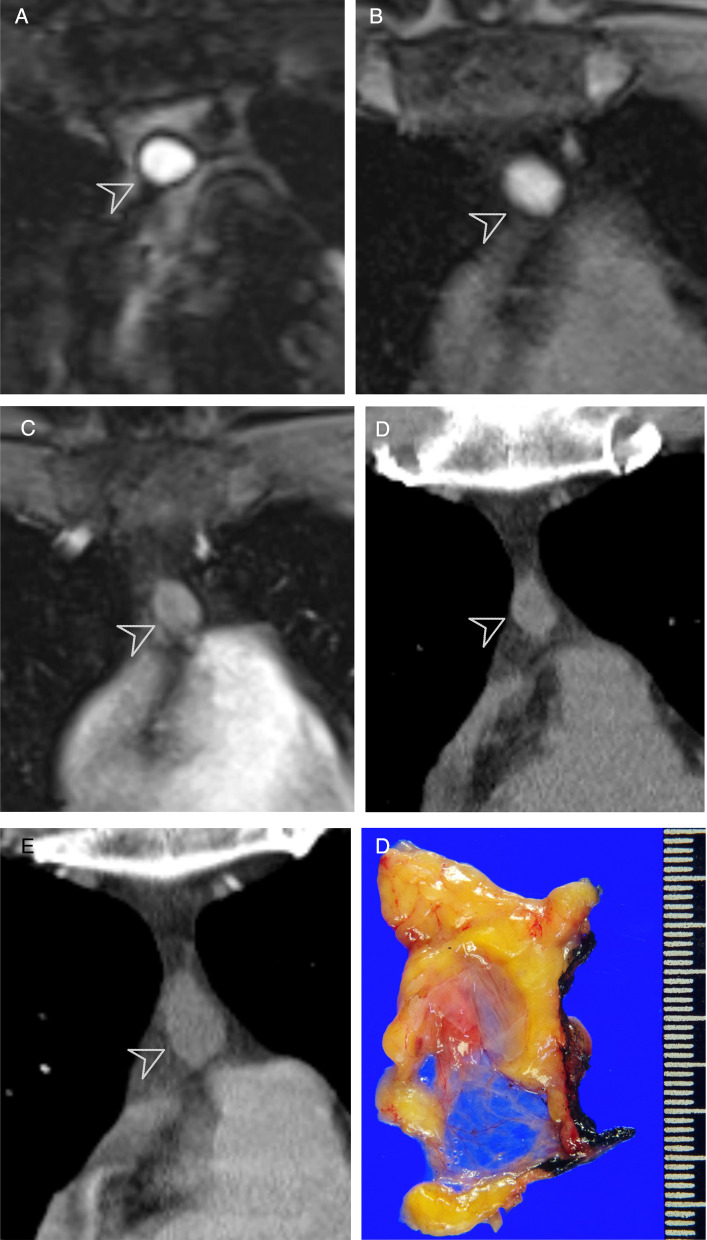
Follow-up outcome of 47-year-old female with incidentally detected anterior mediastinal lesion who underwent chest MRI. On chest MRI, a 19 mm unilocular and ovoid anterior mediastinal lesion shows both high signal intensity on coronal T2 (arrowhead, **a**) and pre-contrast T1-weighted images (arrowhead, **b**) and no remarkable enhancement on post-contrast T1-weighted image (arrowhead, **c**). Patient underwent annual follow-up for the anterior mediastinal lesion. Compared to the baseline non-contrast coronal chest CT image (arrowhead, **d**), the lesion size increase from 15 to 25 mm on follow-up coronal contrast-enhanced chest CT image after 3 years (arrowhead, **e**). Patient underwent thymectomy, and the gross pathology shows a 25-mm well-defined ovoid cyst with cystic content of mucinous material (**f**). The pathologic diagnosis was a thymic cyst

Most cysts (75.3%, 140/186) showed unilocular and nonlobulated shape, but 18.3% (34/186) showed unilocular and lobulated shape, and 6.5% (12/186) had multilocular and lobulated shape. Among the patients with probable cysts, 33.3% (62/186) showed high signal intensity on pre-contrast T1-weighted image, and 24.7% (46/186) showed thin and smooth wall enhancement on MRI (Table 2). Follow-up outcomes of patients with probable cysts on MRI are demonstrated in Fig. 3 and Table 3. In patients with more than 2-year follow-up, the size of lesion unchanged in 39.6% (36/91), decreased in 16.5% (15/91), and fluctuated (decreased and increased, or increased and decreased) in 8.8% (8/91). Among those patients, 35.2% (32/91) showed increase in size during follow-up period and six finally underwent surgical resection which all confirmed benign cystic lesion (Fig. 4). None of the patients showed new enhancing nodular solid portion, aggravated wall thickening with thick enhancement, or septation during follow-up exams. A total of 19 (19/91, 20.9%) patients underwent surgery in patients with probable cysts on MRI and 17 patients were confirmed as a thymic cyst, and two patients were confirmed pericardial cyst on pathology. Confidence level of diagnosis was mostly high (180/186, 96.8%), except six patients (6/186, 3.2%) who had small lesions (median 13 mm; IQR, 11–19) with diffuse high signal intensity on pre-contrast T1-weighted image and they were rated as intermediate confidence level of diagnosis.

**Table 2:** Imaging characteristics of anterior mediastinal cystic lesions on chest MRI

	Probable cyst (*n* = 186)	Indeterminate lesion (*n* = 18)
*Size, mm*^a^	24 (18–33)	39 (22–54)
*Morphologic feature*		
Unilocular and nonlobulated	140 (75.3)	8 (44.4)
Unilocular and lobulated	34 (18.3)	2 (11.1)
Multilocular and lobulated	12 (6.5)	8 (44.4)
*T2-weighted image*		
High signal intensity	186 (100.0)	16 (88.9)
Intermediate or low signal intensity	0 (0)	2 (11.2)
Signal intensity, mean		
Mean^a^	201.8 (138.5–354.2)	163.6 (111.9–208.1)
SD^a^	44.0 (32.2–60.7)	54.1 (32.7–109.7)
*Pre-contrast T1-weighted image*		
High signal intensity	62 (33.3)	13 (72.2)
Low or intermediate signal intensity	124 (66.7)	5 (27.8)
Signal intensity		
Mean^a^	150.0 (99.1–223.5)	163.3 (139.8–262.1)
SD^a^	24.6 (17.2–39.7)	47.5 (18.0–64.5)
*Diffusion-weighted image*		
No restriction	186 (100.0)	14 (77.8)
Diffusion restriction	0 (0)	4 (22.2)
ADC value		
Mean^a^	3.1 (2.7–3.6)	1.8 (1.4–2.5)
SD^a^	0.3 (0.2–0.5)	0.4 (0.2–0.6)
*Contrast-enhanced T1-weighted image*		
No enhancement	140 (75.3)	0 (0)
Thin and smooth wall enhancement	46 (24.7)	3 (16.7)
Eccentric enhancing wall thickening	0 (0)	13 (72.2)
Nodular enhancement	0 (0)	8 (44.4)

Unless indicated otherwise, data are percentages in parentheses

^a^Data are median with interquartile ranges in parentheses

*ADC* apparent diffusion coefficient, *SD* standard deviation

**Table 3:** Follow-up outcome of probable cysts defined on chest MRI with more than 2- or 5-years’ follow-up

	≥ 2 year follow-up (*n* = 91)	≥ 5 year follow-up (*n* = 26)
Unchanged	36 (39.6)	3 (11.5)
Decreased	15 (16.5)	5 (19.2)
Fluctuated^a^	8 (8.8)	7 (26.9)
Increased	32 (35.2)	11 (42.3)
Size increase → Surgical resection	6 (6.6)	1 (3.8)

Data are percentages in parentheses

^a^Fluctuated refers to lesions that change in size in both directions (decreased and increased or increased and decreased) during follow-up periods

### Imaging characteristics and outcome of indeterminate lesions on chest MRI

**Fig. 5:**
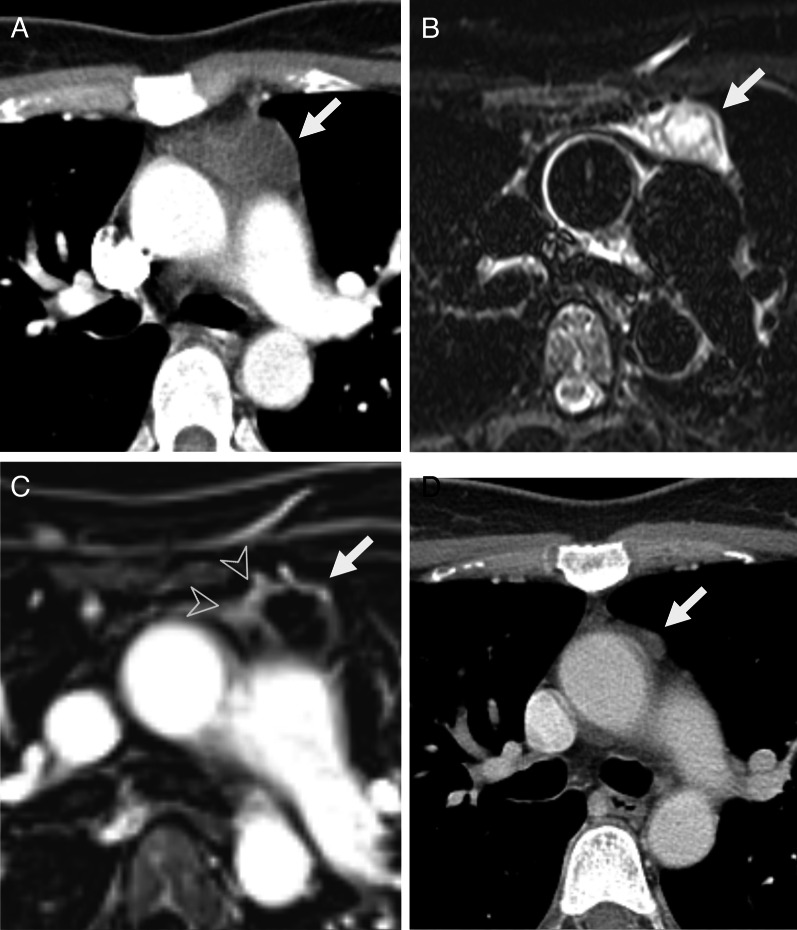
Follow-up outcome of 46-year-old female with anterior mediastinal cystic lesion who underwent chest MRI. Initial contrast-enhanced chest CT shows a 46 mm anterior mediastinal mass with heterogeneous attenuation (arrow, **a**). Patient underwent chest MRI after 2 weeks. On chest MRI, the lesion shows mostly high signal intensity with linear dark signal intensity septa on axial T2-weighted images (arrow, **b**), suggesting multilocular cystic lesion. On subtraction images of axial post- and pre-contrast T1-weighted images, the lesion shows enhancing wall (arrow, **c**) with irregular and eccentric wall thickening (arrowhead, **c**). The lesion can be classified as an indeterminate lesion (complex cyst) based on chest MRI findings and the differential diagnosis of MRI reading included complicated thymic cyst and cystic thymoma. Patient underwent 3-month follow-up after the MRI rather than immediate surgery. On 3-month follow-up CT, the lesion shows a marked decrease in size of anterior mediastinal lesion (arrow, **d**), which suggests the benign nature of lesion, complicated thymic cyst

Among the 18 patients classified as indeterminate lesion on MRI, eccentric thickening or thick wall was noted in 13 (72.2%) and nodular enhancement was noted in eight (44.4%) (Table 2). The lesion size was significantly larger than that of the lesions classified as probable cysts on MRI (median 39 vs. 24 mm; *p* = 0.02). In patients with an indeterminate lesion on MRI, 8 patients underwent surgery. Among them, thymoma in three patients (3/8, 37.5%), teratoma in one patient (1/8, 12.5%) were confirmed. Four of the eight remaining patients (4/8, 50%) had thymic cysts. On the histopathological examination of lesions proven to be thymic cysts, all lesions appeared as multilocular thymic cysts with solid portion and/or sebaceous material or demonstrated lymphoid hyperplasia. Among the 10 patients who did not undergo surgery, the size of lesion markedly decreased or nearly regressed (longitudinal size < 10 mm) in four patients on follow-up examinations, suggesting the benign nature of lesion (Fig. 5). The median follow-up interval between the two examinations that showed a marked decrease in size was 3.4 months (IQR, 2.7–4.2). Finally, in patients with an indeterminate lesion on MRI, 16.7% (3/18) were confirmed thymoma and 44.4% (8/18) were confirmed cysts.

### Follow-up strategies in clinical practice after chest MRI

In patients with probable cyst except who underwent immediate surgery after chest MRI and underwent follow-up solely for anterior mediastinal cystic lesion (*n* = 142), the follow-up duration after MRI scan was 1.0 years (IQR: 0–2.0 years; range, 0–10.7 years). Among the patients with probable cysts on MRI, 38.7% (55/142) of patients did not underwent further follow-up after MRI. Among 87 patients who underwent follow-up after MRI, 59.8% (52/87) underwent follow-up for up to 2 years. The median follow-up interval between first imaging follow-up after MRI and baseline MRI was 12 months (IQR, 6–13), which was variable among six physicians (median 12 months for physician 1–3 from institution A; 6, 4, and 9 months for physician 4, 5, and 6 from institution B; range 1–34 months; *p* = 0.08) and institutions (median 12.0 month in institution A vs. 6 months in institution B; *p* = 0.01). Proportion of patients without further follow-up after MRI was also different among the physician (50.0% for physician 1, 31.6% for physician 2; 40.0% for physician 3 from institution A; 0% for physician 4; 0% for physician 5; 60.0% for physician 6 from institution B; *p* = 0.007). The number of follow-up imaging after MRI was different across the physicians (*p* = 0.001) and institutions (median follow-up numbers, 1 in institution A vs. 3 in institution B; *p* < 0.001).

In patients with indeterminate lesion except those who underwent immediate surgery after chest MRI and underwent follow-up solely for anterior mediastinal cystic lesion (*n* = 9), five patients underwent neither further follow-up imaging nor surgery after chest MRI and five patients underwent follow-up with median 1.1 years (IQR: 0.3–1.4 years; range, 0.3–1.8 years) of total follow-up duration after MRI. Follow-up interval between first imaging follow-up after MRI and baseline MRI was median 6.2 months (IQR: 3.0–12.1 months; range, 1.4–17.2 months). The proportion of patients who underwent first follow-up after chest MRI with follow-up intervals of less than 3 months was higher in those with indeterminate lesion than in those with probable cysts on chest MRI (indeterminate lesion vs. probable cysts, 40.0% vs. 2.3%; *p* = 0.01).

## Discussion

Chest MRI is a widely accepted modality for evaluation of anterior mediastinal lesions. However, clinical outcomes and management of anterior mediastinal cystic lesions diagnosed on MRI is not clearly determined. In our study, 91% of patients were classified as probable cysts among the patients with anterior mediastinal cystic lesions demonstrated on MRI and none of those lesions turned out to be tumor despite changes in size during follow-up. In patients with indeterminate lesions on MRI, 44% had pathologically proven thymic cysts or showed marked decrease in size on follow-up images, suggesting the benign nature of the lesion. Despite varying follow-up intervals according to physicians, most patients with probable cysts were followed up for up to 2 years after chest MRI.

In our study, diagnosing anterior mediastinal cystic lesions using MRI was reliable and accurate for excluding malignancy. All cystic lesions classified as probable cysts on MRI showed no new features suggesting malignant transformation during follow-up such as irregular wall thickening or mural nodules. The follow-up outcome of probable cysts defined by chest MRI in our study were concordant with the findings of Ackman et al., which included only unilocular cysts. Probable cysts in anterior mediastinum diagnosed based on MRI showed a variable change in size on follow-up (decrease, increase or fluctuate) [7]. This is well known and the reason for variable size change in the thymic cysts, can be chronic and recurrent internal hemorrhage and resorption of the internal materials [7, 12]. However, increase in size of theses lesions usually raises concern for malignancy in routine practice. Indeed, in our study, six cystic lesions that were probable thymic cysts based on MRI, were resected due to increase in size. On the basis of our study and Ackman et al., in this situation, follow-up imaging should be considered rather than immediate surgical resection. Furthermore, in case of probable cysts on chest MRI, follow-up may not be necessary.

About 10% of anterior mediastinal cystic lesions were classified as indeterminate. Most lesions showed either of eccentric enhancing wall thickening or nodular enhancement on MRI. For the indeterminate lesion classified based on chest MRI, the differential diagnoses include cystic thymoma, teratoma, or complicated thymic cysts. Actually, lesions cannot be clearly differentiate based on imaging findings, as both benign and malignant disease can demonstrate irregular, eccentric thickening of wall and/or nodular enhancement. These lesions should be followed or resected, depending upon the level of radiologic (i.e., morphology and size of lesion) and clinical suspicion. Interestingly, 40% of patients with indeterminate lesion but who did not undergo surgery, showed marked decrease in size of lesion after median 4 months follow-up, suggesting that the lesions were complicated cysts rather than malignant tumors. Although the number of indeterminate lesions in our study was small, this might enhance the possibility of a more conservative approach even for indeterminate lesions. Therefore, deciding whether to perform surgery after a short follow-up may be considered as a management option in these patients.

Pathologic examinations of the indeterminate lesions proven to be thymic cysts in our study demonstrated histopathologic features of multilocular cysts with a solid portion and/or sebaceous material or lymphoid hyperplasia. Multilocular thymic cysts are thought to be the result of an acquired inflammatory process. Severe acute and chronic inflammation causes cholesterol granuloma formation, fibrovascular proliferation, hemorrhage, necrosis, and/or reactive lymphoid hyperplasia with prominent germinal centers [16, 17]. Those acute and chronic inflammation with lymphoid hyperplasia, granuloma formation or fibrovascular proliferation might present as a solid portion or eccentric nodular thickening on MRI.

Regarding follow-up strategies in patients diagnosed as probable cyst after MRI, it was quite variable according to physicians and institutions. This stems from a different degree of concerns and reliability for chest MRI diagnosing anterior mediastinal cystic lesions differentiating benign cyst from cystic degeneration of malignant tumor among physicians. We observed no adverse outcome (malignant transformation or false negative) of the lesions classified as a probable cyst on MRI. Considering that non-complicated or simple cystic lesions of the other organs including kidney and liver do not usually require follow-up [18–20], longer routine follow-up for probable cystic lesions may also not be needed. As chest MRI significantly reduced the number of planned surgical interventions owing to preoperative diagnosis of cysts [21], it may reduce unnecessary subsequent follow-up examinations for an indeterminate thymic lesion at CT. We believe our results can be evidence for building further guideline for management of anterior mediastinal lesions.

Our study had limitations. First, there was no pathologic confirmation of benignity of all probable cysts demonstrated on MRI. Second, the lesion size was measured and compared using not volumetric measures, but maximum longitudinal diameter on the single best plane among axial, coronal, and sagittal images. As the shape of cysts can change, volumetric analysis may be a good way to accurately show the changes between examinations; however, single measurement of maximum diameter can be a more practical way and is mostly used in the other follow-up guidelines, including renal cysts or pancreatic cystic lesions [22]. Third, follow-up imaging examinations after chest MRI were mostly performed with chest CT, except for two patients; therefore, the evaluation of subtle changes regarding internal characteristics of cystic lesions might be limited in our study. However, overt morphologic changes, which should be certainly alert for malignant tumors, can also be detected on CT.

## Conclusion

Diagnosing anterior mediastinal cysts using MRI is reliable. MRI-based management of anterior mediastinal lesions may reduce the number of unnecessary follow-ups and surgeries.

## Supplementary Information

**Additional file 1.** **Table S1.** Representative MRI protocols in institution A. **Table S2.** Representative MRI protocols in institution B.

## Data Availability

The datasets generated or analyzed during the study are available from the corresponding author on reasonable request.

## Abbreviations

ADC: Apparent diffusion coefficient
DWI: Diffusion-weighted images
ITMIG: International Thymic Malignancy Interest Group
MRI: Magnetic resonance imaging
