# Supplementary material for: Characteristics and outcomes of anterior mediastinal cystic lesions diagnosed on chest MRI: implications for management of cystic lesions
Source: Insights Imaging. 2022 Aug 17;13:136. doi: 10.1186/s13244-022-01275-8 (PMC9385942; doi:10.1186/s13244-022-01275-8)
Supplement: Supplementary file 1 — Additional file 1. Table S1. Representative MRI protocols in institution A. Table S2. Representative MRI protocols in institution B. [file 13244_2022_1275_MOESM1_ESM.docx]

**Table S1.** Representative MRI protocols in institution A

|  | Plane | TR/TE | Flip angle | FOV | Slice thickness, mm | Matrix size |
| --- | --- | --- | --- | --- | --- | --- |
| Manufacturer: Siemens |  |  |  |  |  |  |
| Breath-hold ECG-gated T2-weighted turbo spin echo images with double inversion recovery (STIR) | Axial | 1958-2521/65 | 180 | 300 × 300 | 3 | 320 × 208 |
|  | Coronal | 2012-2668/65 | 180 | 300 × 300 | 3 | 320 × 208 |
| Diffusion-weighted images (b0, b500, and b1000) | Axial | 4700-7600/104 | 90 | 240 × 300  256 × 300  272 × 300 | 3 | 150 × 120  150 × 128  150 × 136 |
| Precontrast breath-hold three-dimensional gradient-echo fat-saturated T1-weighted images (Dixon-VIBE) | Axial | 5.4/2.5-3.7 | 9 | 300 × 400  407 × 450 | 2 | 320 × 204  320 × 246 |
| Postcontrast breath-hold three-dimensional gradient-echo fat-saturated T1-weighted images (Dixon-VIBE) | Axial | 4.2-5.4/1.3-3.7 | 9 | 300 × 400  407 × 450 | 2 | 320 × 204  320 × 246 |
|  | Coronal | 4.2-5.4/1.3-2.5 | 9 | 344 × 380  407 × 450 | 2 | 320 × 246 |
|  | Sagittal | 4.2-5.4/1.3-2.5 | 9 | 400 × 400  450 × 450 | 2 | 320 × 256  320 × 272 |

FOV, field of view; ECG, electrocardiography; STIR, short tau inversion recovery; TE echo time; TR, repetition time; VIBE, 3D volumetric interpolated breath-hold examination.

**Table S2.** Representative MRI protocols in institution B

|  | Plane | TR/TE | Flip angle | FOV | Slice thickness, mm | Matrix size |
| --- | --- | --- | --- | --- | --- | --- |
| Manufacturer: Siemens |  |  |  |  |  |  |
| Breath-hold ECG-gated T2-weighted turbo spin echo images with double inversion recovery (STIR) | Axial | 1505-1772/51 | 180 | 312 × 400 | 3 | 320 × 187 |
|  | Coronal | 1589-1810/51 | 180 | 375 × 400 | 3 | 320 × 225 |
| Breath-hold T2-weighted fat-saturated single-shot fast spin echo images (HASTE) | Axial | 360-700/72 | 155-160 | 325 × 400 | 5 | 256 × 166 |
| Breath-hold diffusion-weighted images (b0, b100, and b700) | Axial | 9100/45 | 90 | 286 × 399 | 5 | 134 × 96 |
| Precontrast breath-hold three-dimensional gradient-echo fat-saturated T1-weighted images (Dixon-VIBE) | Axial | 3.35/1.3 | 9 | 312 × 399 | 3 | 384 × 240 |
| Postcontrast breath-hold three-dimensional gradient-echo fat-saturated T1-weighted images (Dixon-VIBE) | Axial | 3.35/1.3 | 9 | 312 × 399 | 3 | 384 × 240 |
|  | Coronal | 3.35/1.3 | 9 | 374 × 399 | 3 | 384 × 288 |

FOV, field of view; ECG, electrocardiography; HASTE, Half-Fourier-acquisition single-shot turbo spin echo; STIR, short tau inversion recovery; TE echo time; TR, repetition time; VIBE, 3D volumetric interpolated breath-hold examination.
